# Intestinal Spontaneous Intramural Hematoma Secondary to Anticoagulation Therapy: A Case Report

**DOI:** 10.7759/cureus.37257

**Published:** 2023-04-07

**Authors:** Karim El Aidaoui, Wahib Lahlou, Abderrahim Bourial, Nawal Bouknani, Chafik El Kettani

**Affiliations:** 1 Anesthesia and Critical Care, Cheikh Khalifa International University Hospital, Mohammed VI University of Health Sciences (UM6SS), Casablanca, MAR; 2 Otolaryngology, Head and Neck Surgery, Cheikh Khalifa International University Hospital, Mohammed VI University of Health Sciences (UM6SS), Casablanca, MAR; 3 Radiology, Cheikh Khalifa International University Hospital, Mohammed VI University of Health Sciences (UM6SS), Casablanca, MAR

**Keywords:** spontaneous intramural hematoma, case report, anticoagulation therapy, intestinal obstruction, hematoma

## Abstract

A common complication of anticoagulation therapy is bleeding, especially in patients receiving long-term vitamin K antagonists. Spontaneous intramural hematoma is a rare etiology among life-threatening major bleeds.

An 80-year-old female patient presented with diffuse abdominal pain. Her history included ischemic heart disease and chronic atrial fibrillation treated with 3 mg of acenocoumarol per day. Three days before her admission, she developed diffuse abdominal pain with fecaloid vomiting, bloating, and not passing gas. Palpation of the abdomen revealed asymmetrical distension and pain, with no signs of peritoneal irritation or bleeding. Investigations showed anemia with a hemoglobin level of 9.2 g/dL, a white blood cell count of 14200/mm^3^, a C-reactive protein of 112.6 mg/L, and a prothrombin time of 75.1 seconds with an international normalized ratio (INR) of 8.5. Abdominal contrast-enhanced computed tomography (CT) showed segmental parietal thickening, luminal narrowing, and partial small bowel obstruction secondary to an intramural jejunum hematoma responsible for a gallbladder occlusion with infiltration of the mesenteric fat in front. The patient recovered two days after conservative treatment.

In this case, we report an unusual small bowel intramural hematoma of the jejunum secondary to anticoagulant therapy. Physicians should be aware of this unusual cause of abdominal pain. Early diagnosis may avoid unnecessary surgical exploration.

## Introduction

Bleeding is the most common and serious complication of anticoagulation therapy. Approximately 10%-17% of patients receiving long-term vitamin K antagonists will bleed at least once a year [1]. Spontaneous intramural hematoma is a rare etiology among life-threatening major bleeds. It's defined as the presence of blood between submucosal and muscular layers that may lead to partial or complete bowel obstruction. They have been described in almost every area of the gastrointestinal tract, from the esophagus to the sigmoid colon [2].

In this case, we report an unusual small bowel intramural hematoma of the jejunum secondary to anticoagulant therapy. Front-line physicians should be aware of this unusual cause of abdominal pain. Early diagnosis may avoid unnecessary surgical exploration. The treatment is conservative in the vast majority of cases [3].

## Case presentation

An 80-year-old female patient presented to the emergency unit with diffuse abdominal pain. Her medical history included ischemic heart disease and chronic atrial fibrillation, both treated with 3 mg of acenocoumarol per day. Three days before her admission, she developed diffuse abdominal pain with fecaloid vomiting, bloating, and not passing gas.

On admission, her temperature was 37.5 °C, her pulse was 104 beats per minute, her blood pressure was 130/60 mmHg, her respiratory rate was 18 breaths per minute, her oxygen saturation was 96% in ambient air, and she had a Glasgow Coma Scale of 15/15.

An abdominal physical examination revealed asymmetrical distension and pain upon palpation but no signs of peritoneal irritation. Similarly, a rectal examination showed no signs of bleeding and no hernia orifices. The rest of the physical examination was normal.

The biological assessment showed anemia with a hemoglobin level of 9.2 g/dL, a white blood cell count of 14200/mm3 with 86% segmented neutrophils, a C-reactive protein of 112.6 mg/L, a pro-calcitonin level of 0.43 ng/ml, serum urea nitrogen of 1.38 g/L, serum creatinine of 29.5 mg/L, sodium of 135 mEq/L, potassium of 5.2 mEq/L, and a prothrombin time of 75.1 seconds with an international normalized ratio (INR) of 8.5 (Table 1).

**Table 1:** Laboratory findings on admission. "( )" indicate the normal range.

Biological parameters	Value (normal range)
White blood cells (/mm^3^)	14200 (4000 - 10000)
Hemoglobin (g/dl)	9.2 (12 - 16)
C-reactive protein (mg/L)	112.6 (< 6)
Procalcitonin (ng/ml)	0.43 (< 0.1)
Serum urea nitrogen (g/L)	1.38 (0.18 - 0.45)
Serum creatinine (mg/L)	29.5 (5 - 12)
Sodium (mEq/L)	135 (135 - 145)
Potassium (mEq/L)	5,2 (3.6 - 5)
Prothrombin time (seconds)	75.1 (10 - 13)
International normalized ratio (INR)	8.5 (0.8 - 1.1)

**Figure 1:**
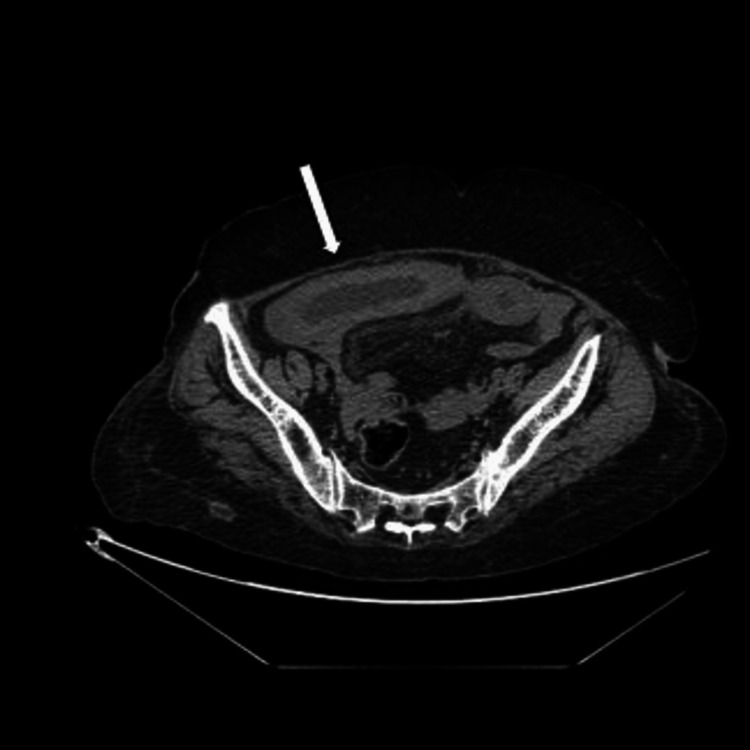
A CT of the abdomen shows circumferential parietal thickening of the grelic gloves.

**Figure 2:**
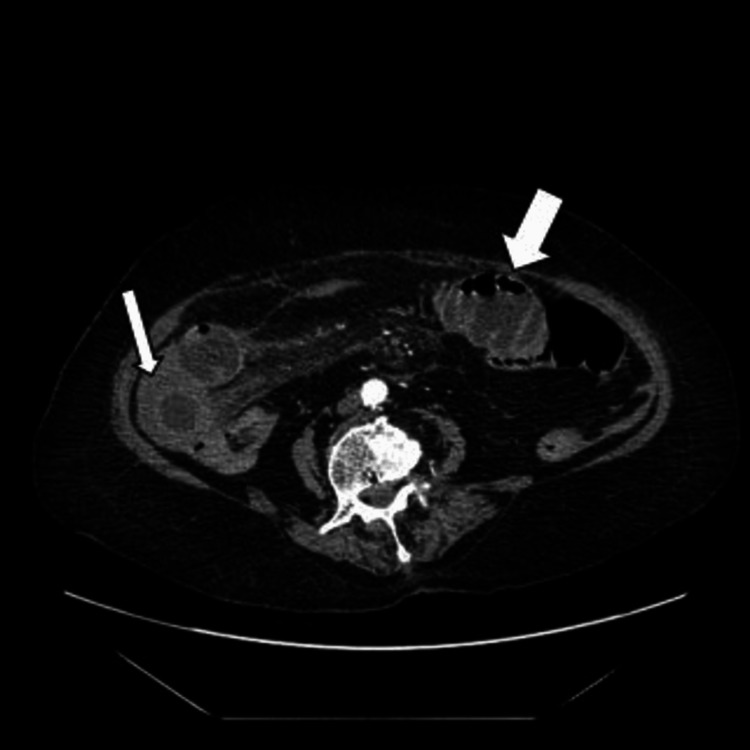
A contrast CT shows a parietal hematoma (narrow arrow) responsible for a gallbladder occlusion (wide arrow) with the infiltration of the mesenteric fat in front.

**Figure 3:**
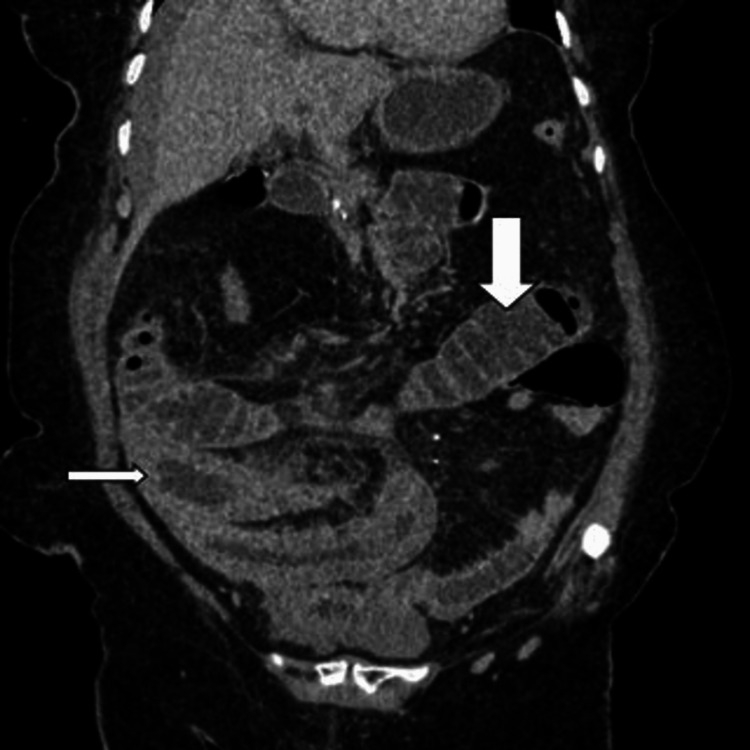
A contrast CT in a coronal slice shows a parietal hematoma (narrow arrow) responsible for a bowel obstruction (wide arrow) with the infiltration of the mesenteric fat with reactionary circumferential parietal thickening of some grelic gloves.

Abdominal contrast-enhanced computed tomography (CT) showed segmental parietal thickening, luminal narrowing, and partial small bowel obstruction secondary to an intramural jejunum hematoma responsible for a gallbladder occlusion with infiltration of the mesenteric fat in front (shown in Figures 1-3).

The diagnostic hypothesis was that the small bowel obstruction was secondary to an intramural hematoma because of an anticoagulant overdose.

The patient was treated with parenteral vitamin K, four units of fresh frozen plasma (FFP), and a transfusion of one packed red blood cell. Equally important, we undertook parenteral hydration, nasogastric decompression, discontinuation of acenocoumarol, and digestive bowel resting.

The patient recovered two days after conservative treatment. We noted a reduction in abdominal distension and the resumption of transit. On the third day, the patient resumed oral alimentation. Hence, anticoagulation therapy was restarted on the fifth day with an adapted dose. The patient preferred not to use non-vitamin K antagonist oral anticoagulant (NOAC) therapy because of its high price. She left the hospital after seven days.

## Discussion

Described in 1838 [4] and reported since the 1960s [5], intramural small bowel hematoma is a rare disease with an incidence of one case per 2500 patients [6, 7]. The most common area involved in localization is the jejunum, followed by the ileum and the duodenum [3].

Spontaneous intramural hematomas of the gastrointestinal tract (SIHGT) are usually observed after abdominal trauma. Many non-traumatic causes can lead to SIHGT, such as anticoagulant therapy, hemophilia, Von Willebrand disease, idiopathic thrombocytopenic purpura, leukemia, lymphoma, vasculitis, collagenosis, chemotherapy, bone marrow transplantation, liver diseases, peptic ulcers, and protein C and S synthesis disorders [2]. Among non-traumatic etiologies, anticoagulation therapy is considered the main etiology of intramural hematoma of the digestive tract [2].

SIHGT can present itself through a wide spectrum of symptoms. It can vary from mild, vague abdominal pain to acute abdomen presentation. Common presenting symptoms are abdominal pain, nausea and vomiting, gastrointestinal hemorrhage, and signs of peritoneal irritation or intestinal obstruction [7,8].

A CT scan is the most commonly performed imaging test for SIHGT diagnosis. A non-contrast CT should be performed before an oral or intravenous contrast CT [9]. In fact, contrast CT intestinal wall enhancement can disguise an intramural hematoma. Signs to look for are circumferential wall thickening, intramural hyperdensity, luminal narrowing, and intestinal obstruction [7].

An abdominal ultrasound can show a thickened intestinal wall, mainly involving the submucosal layer. This test alone isn’t specific, but when combined with a CT scan, it is efficient [7,10].

Vitamin K antagonists are widely used drugs around the world. They make up the main therapeutic arsenal for thromboembolic complications of atrial fibrillation with direct-acting oral anticoagulants. Acenocoumarol and warfarin are vitamin K antagonist drugs that prevent the action of vitamin K-dependent factors (II, VII, IX, and X) by inhibiting gamma-carboxylation. These drugs have a tight therapeutic index and a labile interindividual response. Several foods and drugs can interact with vitamin K antagonists and alter their effects [11].

Patients treated with vitamin K antagonists require frequent INR monitoring. Levels above the therapeutic range increase the risk of bleeding. However, bleeding may also occur in patients with a correct INR target [12,13].

Shredding of terminal arteries appears to be the most plausible physiopathological explanation of SIHGT. After the vascular rupture, blood leaves the mesentery and infiltrates the intestinal wall muscle. Thus, the hematoma dissects its way between the intestinal mucosa and the muscle layer. Therefore, the viability of the mucosa is preserved, unlike in mesenteric vascular occlusion [2,14].

All digestive tracts can be affected. Jejunum makes up the most commonly affected part in non-traumatic intramural hematomas of the gastrointestinal tract [15-16].

Medical treatment is preferred in the management of SIHGT unless there are signs of peritonitis. The first step is to discontinue anticoagulant treatment and oral food intake (digestive rest) with nasogastric tube stomach decompression. Vitamin K administration and prothrombin complex concentrates (PCC) can enhance hemostasis [17]. We can consider blood transfusions for patients with severe anemia. A surgical approach can prevail in cases of peritonitis because of ischemia or perforation. In the same way, a severe intra-abdominal hemorrhage or refractory gastrointestinal blockage can require surgery [15-18].

## Conclusions

The most common side effects of vitamin K antagonists are hemorrhagic accidents. Close monitoring when using those drugs is required to prevent serious hemorrhagic accidents. Intestinal spontaneous intramural hematoma is a rare hemorrhagic accident caused by vitamin K antagonists. Physicians should be aware of this unusual presentation of intestinal obstruction in order to avoid unnecessary surgical exploration.
